# Expression Characteristics of Odorant Receptor Gene *AcerOR120* and Its Physiological Roles in Mediating Odor Recognition in *Apis cerana cerana*

**DOI:** 10.3390/insects17090967

**Published:** 2026-09-19

**Authors:** Jinjia Liu, Bailu Xie, Shiyan Jia, Yu Gong, Miaomiao Liu, Huiting Zhao

**Affiliations:** 1 College of Basic Medicine, Changzhi Medical College, Changzhi 046000, China; 2 College of Life Science, Shanxi Agricultural University, Jinzhong 030801, China

**Keywords:** *Apis cerana cerana*, odorant receptor gene, bioinformatic analysis, spatiotemporal expression, RNA interference, electroantennogram

## Abstract

This study aims to characterize the structural features and spatiotemporal expression patterns of the protein encoded by odorant receptor gene *AcerOR120*, and to explore its biological functions in olfactory recognition in *Apis cerana cerana*. Bioinformatic software was used to predict the transmembrane topology, secondary/tertiary structures and conserved domains of the AcerOR120 protein. A neighbor-joining phylogenetic tree was constructed based on homologous sequences within the genus Apis. qRT-PCR was performed to detect the gene expression profiles in antennae of worker bees at 1, 5, 10, 15, 20 and 25 days old, as well as different tissues of foragers. EAG recording was applied to compare the electrophysiological responses of worker antennae to 11 volatile odorants before and after gene silencing via RNA interference. The open reading frame of *AcerOR120* is 1203 bp, encoding a 401-amino-acid protein containing seven transmembrane domains with an intracellular N-terminus. Its secondary structure is dominated by α-helices accounting for 82.79%, which matches the typical structural signature of insect odorant receptors. Phylogenetic analysis revealed that AcerOR120 shares high conservation across *Apis* species. Spatiotemporal expression assays showed that transcript abundance in antennae increased first and then decreased with worker age, reaching the minimum at day 1 and the maximum at day 20. Among tissues of foragers, the highest expression was detected in antennae and thoraxes, followed by heads and legs, while abdomens and wings possessed low expression levels. RNAi silencing significantly reduced overall EAG responses of antennae to all 11 tested odorants, with the most dramatic decreases observed for Geraniol, Decanal, Ocimene and (+)-Dihydrocarvone. *AcerOR120* encodes a canonical insect odorant receptor and is highly expressed in forager antennae. It mediates the recognition of multiple floral volatiles and pheromones and is speculated to participate in olfactory-related physiological processes, including nectar source localization and social behavior regulation in *A. cerana cerana*.

## Simple Summary

The keen olfactory capacity of *Apis cerana cerana* provides substantial plasticity in pollination and resource selection, with odorant receptors (ORs) playing indispensable roles in detecting and transducing environmental chemical signals. In this study, we systematically characterized the structural properties, evolutionary conservation, and spatiotemporal expression patterns of *AcerOR120*, and evaluated alterations in olfactory sensitivity following gene knockdown. The protein exhibits a classical seven-transmembrane architecture, shares high sequence conservation with orthologs across the genus *Apis*, and peaks in expression during the early foraging stage (15- and 20-day-old workers), predominantly localized in antennae, heads, thoraces, and legs. Silencing *AcerOR120* via RNA interference significantly weakened antennal sensitivity to key odorants, including geraniol, isoamyl acetate, decanal, (*E*)-2-octenal, ocimene, linalool, and (+)-dihydrocarvone. These results demonstrate that *AcerOR120* is involved in foraging behavior by modulating the perception of floral scents and colonial pheromones, offering a functional molecular target for deciphering and manipulating olfactory recognition in *A. c. cerana*.

## 1. Introduction

Insects rely on a highly specialized and extremely sensitive olfactory system to accurately identify odorants of various origins in their surrounding environment. This system is crucial for a series of life activities such as locating food, avoiding predators, mating, and oviposition, and it is essential for their survival and reproduction [1,2,3]. The recognition of environmental odorants involves a complex process that includes multiple proteins such as odorant-binding proteins (OBPs), chemosensory proteins (CSPs), odorant receptors (ORs), ionotropic receptors (IRs), sensory neuron membrane proteins (SNMPs), and odorant-degrading enzymes [4,5,6]. Among these, ORs are key components of the olfactory system, playing important roles in detecting odor molecules and transducing olfactory signals downstream. The conversion of chemical signals into electrical signals, mediated by ORs on the dendritic membranes of olfactory sensory neurons, is the basis of insect olfactory perception [7,8]. Insect ORs can be structurally divided into two types: one is the highly conserved and broadly expressed odorant receptor co-receptor (Orco), and the other is the conventional ORs, which are numerous but share low homology within or between species; they form heteromeric complexes with Orco to execute olfactory recognition functions [9].

Protein structure is the basis of its function, and its precise determination relies mainly on experimental instrumental analysis and computational model prediction. However, for large membrane-embedded complexes such as odorant receptors, isolated detection makes it difficult to guarantee structural integrity and functionality, so cryo-electron microscopy has become the preferred method for observing their overall structure in the native membrane environment [10]. But due to the high cost and complex operation, only a few insect OR structures have been resolved so far. Therefore, most OR analyses currently rely on computational model predictions, which are rapidly developing with the continuous optimization of algorithms, big-data processing capabilities, and deep-learning levels, and the accuracy of predictions is steadily improving. Nevertheless, it should be emphasized that all prediction models are highly dependent on experimentally determined real structures as a foundation; the more and higher-quality experimentally resolved protein structures there are, the more reliable the model predictions become [11].

Electroantennography (EAG) is the most commonly used electrophysiological method for studying olfaction [12]. It records the voltage deflection generated between the two ends (base and tip) of an insect antenna upon odor stimulation, yielding cumulative potential changes from a large population of olfactory sensory neurons. This technique is now widely used to evaluate the function of specific olfactory genes (such as ORs or OBPs). For example, Cao et al. [13] performed RNA interference (RNAi) of *FoccOBP7* in Frankliniella occidentalis and found that the thrips showed significantly reduced EAG responses to dibutyl phthalate, confirming that FoccOBP7 is involved in the binding of dibutyl phthalate.

*Apis cerana cerana* is an indigenous species unique to China. It has advantages such as strong resistance, low consumption, and efficiency in collecting scattered nectar and pollen sources, making it very suitable for rearing in mountainous areas of China and playing an important role in maintaining ecological balance and species diversity [14]. As a social insect with diverse division of labor, *A. c. cerana* relies on varied sensory cues for communication. Odorant receptors (ORs), as a key component of the olfactory system, have been demonstrated to regulate diverse physiological and behavioral processes in this species. Current research has increasingly focused on the molecular level. For instance, ORs participate in hygienic behavior by interacting with highly expressed odorant-binding proteins (OBPs), downregulate their expression to conserve metabolic energy under starvation, and distinguish varied concentrations of floral volatiles to aid in locating pollination resources. These mechanisms collectively enhance the survival and task performance of *A. c. cerana*, highlighting the substantial research value of these receptors [15,16].

In this study, we focused on the odorant receptor gene AcerOR120 identified from previous transcriptomic data. We analyzed and predicted its structure and function using various bioinformatics software and constructed its homologous relationships within the genus Apis. We also analyzed the expression of AcerOR120 in antennae of different developmental stages and in different tissues of foragers by qRT-PCR, and performed EAG detection after silencing *AcerOR120* to preliminarily understand its role in olfactory perception of *A. c. cerana*, with the aim of providing a theoretical basis for further research on the biological functions of this gene. 

## 2. Materials and Methods

### 2.1. Test Insects

The *A. c. cerana* used in this experiment were all collected from the experimental apiary of the College of Animal Science and Technology, Shanxi Agricultural University. Strong, healthy colonies were selected. To establish the spatiotemporal expression profile of OR120, newly emerged worker bees were marked on the thorax with non-toxic paint pens and returned to the hive. Antennae were sampled at 1, 5, 10, 15, 20, and 25 days of age. Additionally, foragers were collected to harvest antennae, heads (without antennae), thoraces, abdomens, legs, and wings. For RNAi assays, foragers were orally administered synthesized dsRNA (8 μg/bee in 50% sucrose solution), with untreated sucrose and dsGFP serving as negative controls; antennae were collected at 12, 24, 48, and 72 h post-feeding to evaluate silencing efficiency. All tissue samples across time points and treatments were collected with three biological replicates (approx. 300 bees each) and analyzed by qRT-PCR.

### 2.2. Main Reagents and Instruments

Total RNA extraction reagent Trizol was purchased from Invitrogen (Carlsbad, CA, USA); cDNA synthesis kit PrimeScript™ RT Reagent Kit with gDNA Eraser (Perfect Real Time) was from TaKaRa (Beijing, China); dsRNA synthesis kit T7 RiboMAX™ Express RNAi System was from Promega (Beijing, China); fluorescent quantitative kit SYBR Premix Ex Taq™ II (Tli RNaseH Plus) was from TaKaRa (Dalian, China); anhydrous ethanol, isopropanol, chloroform and other analytical reagents were from Tianjin Kermel Chemical Reagent Co., Ltd. (Tianjin, China). 

Instruments included: 5810R high-speed refrigerated centrifuge (Eppendorf, Hamburg, Germany), 7500 real-time PCR system (Applied Biosystems, Foster City, CA, USA), NanoDrop 2000c ultra-micro nucleic acid/protein analyzer (Thermo Fisher Scientific, Waltham, MA, USA), and insect electroantennography system (Syntech, Buchenbach, Germany).

### 2.3. Primer Design

Using the online tool Primer3 Plus (version 3.3.0; https://www.primer3plus.com/), primers for qPCR and RNAi experiments were designed based on the transcriptomic sequences. Two dsRNA fragments of different molecular weights (*dsOR120*-1 and *dsOR120*-2) were designed. The reference gene for qPCR was AcerArp1 (HM640276.1) obtained from NCBI, and the control gene for RNAi was the green fluorescent protein gene (GFP) (JQ064510.1) from NCBI (See Table 1 for primer sequences).

### 2.4. Bioinformatic Analysis

Using the translated AcerOR120 amino acid sequence as a template, TMHMM-2.0 (http://www.cbs.dtu.dk/services/TMHMM/, accessed on 10 March 2026) was used to analyze transmembrane topology; PSIPRED (http://bioinf.cs.ucl.ac.uk/psipred/, accessed on 10 March 2026) and Swiss-Model (https://swissmodel.expasy.org/, accessed on 10 March 2026) were used for secondary and tertiary structure predictions, respectively. NCBI BLASTp (https://blast.ncbi.nlm.nih.gov, accessed on 15 March 2026) was used to search for the top 50 homologous sequences with high similarity from the genus Apis. A phylogenetic tree was constructed using the neighbor-joining method in MEGA 11 software (https://www.megasoftware.net, accessed on 16 March 2026), and then visualized and refined using iTOL v6 (https://itol.embl.de, accessed on 18 March 2026), with branches of different species colored differently to distinguish their taxonomic affiliations.

### 2.5. Total RNA Extraction and cDNA Synthesis

Total RNA from each tissue sample was extracted according to the RNA extraction kit instructions. After determining concentration and purity, cDNA was synthesized using the reverse transcription kit and stored at −20 °C for later use or directly used for next steps. cDNA templates from antennae of different ages and from different tissues of foragers were used for qPCR experiments.

### 2.6. dsRNA Synthesis

Using the primer sequences in Table 1, with *A. c. cerana* antenna cDNA as a template, PCR amplification was performed to prepare dsRNA double-stranded sequences for *AcerOR120*. According to the manual, the appropriate reagents were added to 0.2 mL EP tubes, mixed, and placed in a PCR machine at 37 °C for 1 h to synthesize dsRNA; equal volumes of dsRNA for the target gene were mixed and incubated at 70 °C for 10 min. Then, 1 μL of RNase Solution and 1 μL of RQ1 RNase-Free DNase were added per 20 μL system and incubated at 37 °C for 30 min. For every 100 μL system, 10 μL of sodium acetate (pH 5.2) and 100 μL of isopropanol were added, placed on ice for 5 min, and centrifuged at 14,000 rpm for 10 min at 4 °C to obtain a white pellet of dsRNA. The pellet was washed with pre-cooled 70% ethanol and air-dried at room temperature. Finally, dsRNA was resuspended in ddH_2_O, quantified, and stored at −80 °C.

### 2.7. Real-Time Quantitative PCR

The relative expression levels of *AcerOR120* in antennae at different developmental stages and in different tissues of foragers were calculated using 2^−ΔΔCT^ method. *Arp1* was selected as the internal reference gene due to its stable expression across different tissues and developmental stages in *A. cerana cerana*. The amplification efficiencies of all target and reference gene primers were validated using standard curves, and all efficiencies ranged between 90% and 110% (R^2^ > 0.99). Each treatment consisted of three independent biological replicates (*n* = 100 per replicate), with three technical replicates performed for each biological replicate. The experiment was performed according to the instructions of the real-time PCR kit. The reaction system was 15 μL, containing 7.5 μL of 2× SYBR Premix Ex Taq™ II, 0.6 μL each of forward and reverse primers (10 μmol·L^−1^), 0.4 μL of ROX Reference Dye II (50×), 2 μL of cDNA template, and 5.9 μL of sterile water. The cycling protocol was: 95 °C for 30 s; 42 cycles of 95 °C for 5 s, 60 °C for 30 s, and 95 °C for 15 s; followed by 60 °C for 1 min and 95 °C for 15 s.

### 2.8. Electroantennography (EAG)

Eleven volatile substances that were strongly responded to by bee antennae in previous studies were selected(See Table 2 for Odorant materials). After removing the antenna with a scalpel, it was immediately fixed on the two-pronged electrode with conductive gel. Odorant standards were dissolved in liquid paraffin to prepare a final concentration of 300 μg/μL, with liquid paraffin as a blank control. Then, filter paper strips soaked with 10 μL of test solution were placed in EP tubes connected to the gas delivery device. The continuous carrier gas flow rate is 500 mL/min, and the duration of the stimulation pulse is 0.5 s. To eliminate cumulative effects from fatigue or adaptation, the testing order of odorants was randomized, and the stimulation interval was adjusted to 30–60 s to ensure recovery of the antennal response to a stable baseline. Six antennae were tested in parallel for each odorant. The EAG response recordings were analyzed using EAGPro software (Syntech, Buchenbach, Germany). To correct for signal attenuation during recording, relative EAG values were quantified using the following formula:

Relative EAG (%) = [2 × EAG(X)] / [EAG(std1) + EAG(std2)]

where EAG(X) is the peak amplitude in response to the odor stimulus, and EAG(std1) and EAG(std2) are the amplitudes of the liquid paraffin blank controls recorded before and after the odor stimulus, respectively. 

### 2.9. Statistical Analyses

Homologous sequences of AcerOR120 were retrieved using BLASTp, and multiple sequence alignments were performed using MUSCLE. The phylogenetic tree was constructed using the Neighbor-Joining (NJ) method in MEGA11 (https://www.megasoftware.net, accessed on 16 March 2026), with node reliability assessed by 1000 bootstrap replicates.

Data analyses were performed using JASP (version 0.18.x; JASP Team, University of Amsterdam, Netherlands). Differences between two independent groups were analyzed using Student’s *t*-tests. Differences among multiple samples were analyzed using one-way Analysis of Variance with Tukey’s post-hoc test. GraphPad 8.0 (GraphPad Software Inc., La Jolla, CA, USA) was used to visualize the data.

## 3. Results

### 3.1. Structural Prediction of AcerOR120 Protein

**Figure 1:**
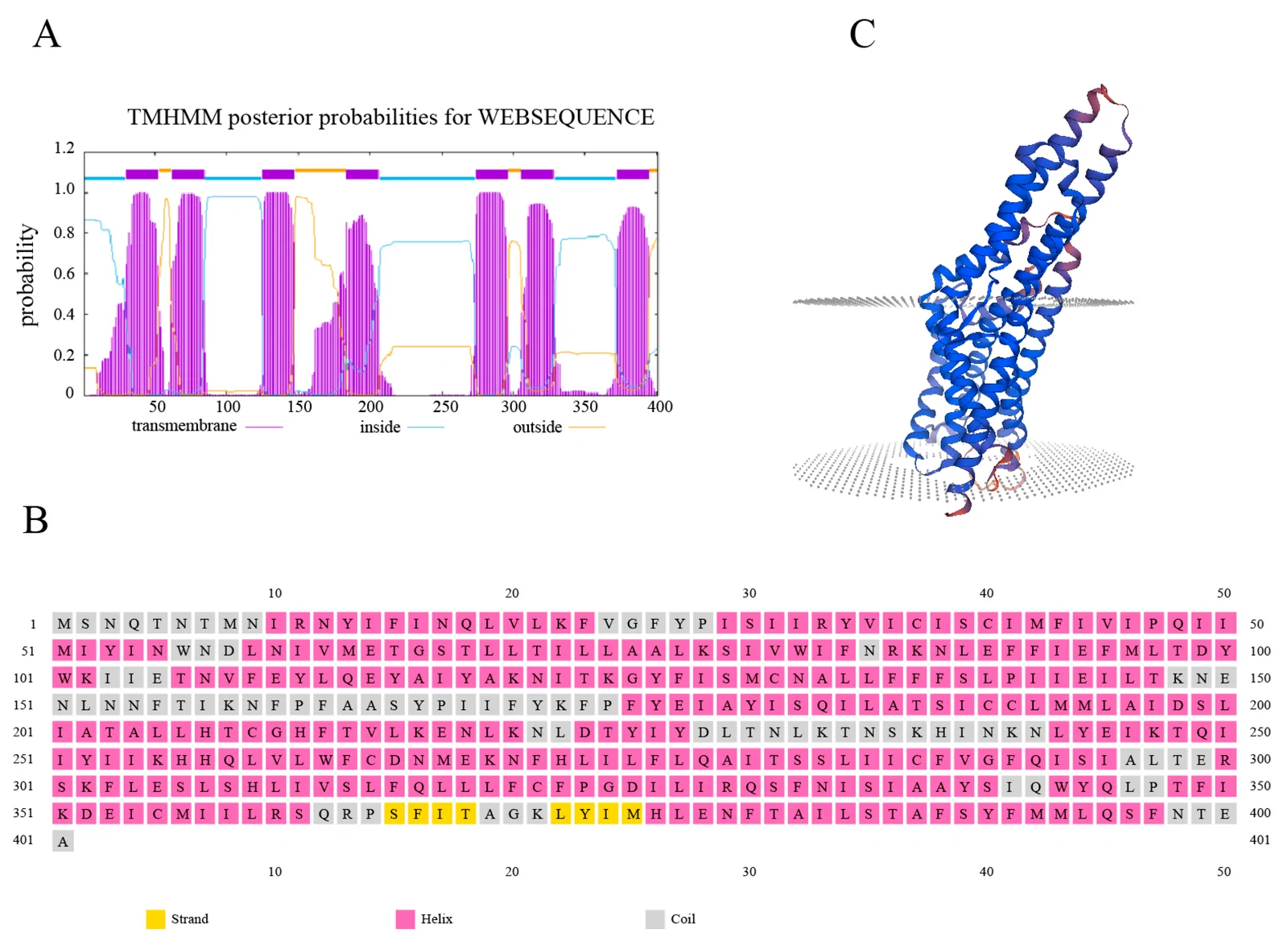
Transmembrane domain (**A**), secondary structure (**B**) and 3-D structure prediction model (**C**) of AcerOR120 protein. In (**B**), pink represents α-helices; Gray represents coils; Yellow represents β-strands.

AcerOR120 protein has 7 transmembrane domains located in the amino acid intervals 32–52, 58–78, 118–138, 172–192, 276–296, 302–322 and 366–386, with the N-terminus located intracellularly (Figure 1A). Secondary structure prediction showed that the protein encoded by AcerOR120 consists of α-helix (Helix), β-strand (Strand) and coil (Coil), with α-helix accounting for 82.79%, coil 15.71%, and β-strand 1.50%. The tertiary structure prediction further confirmed the presence of 7 transmembrane helices and the abundance of α-helices predicted in the secondary structure (Figure 1C).

### 3.2. Phylogenetic Tree Analysis of AcerOR120

**Figure 2:**
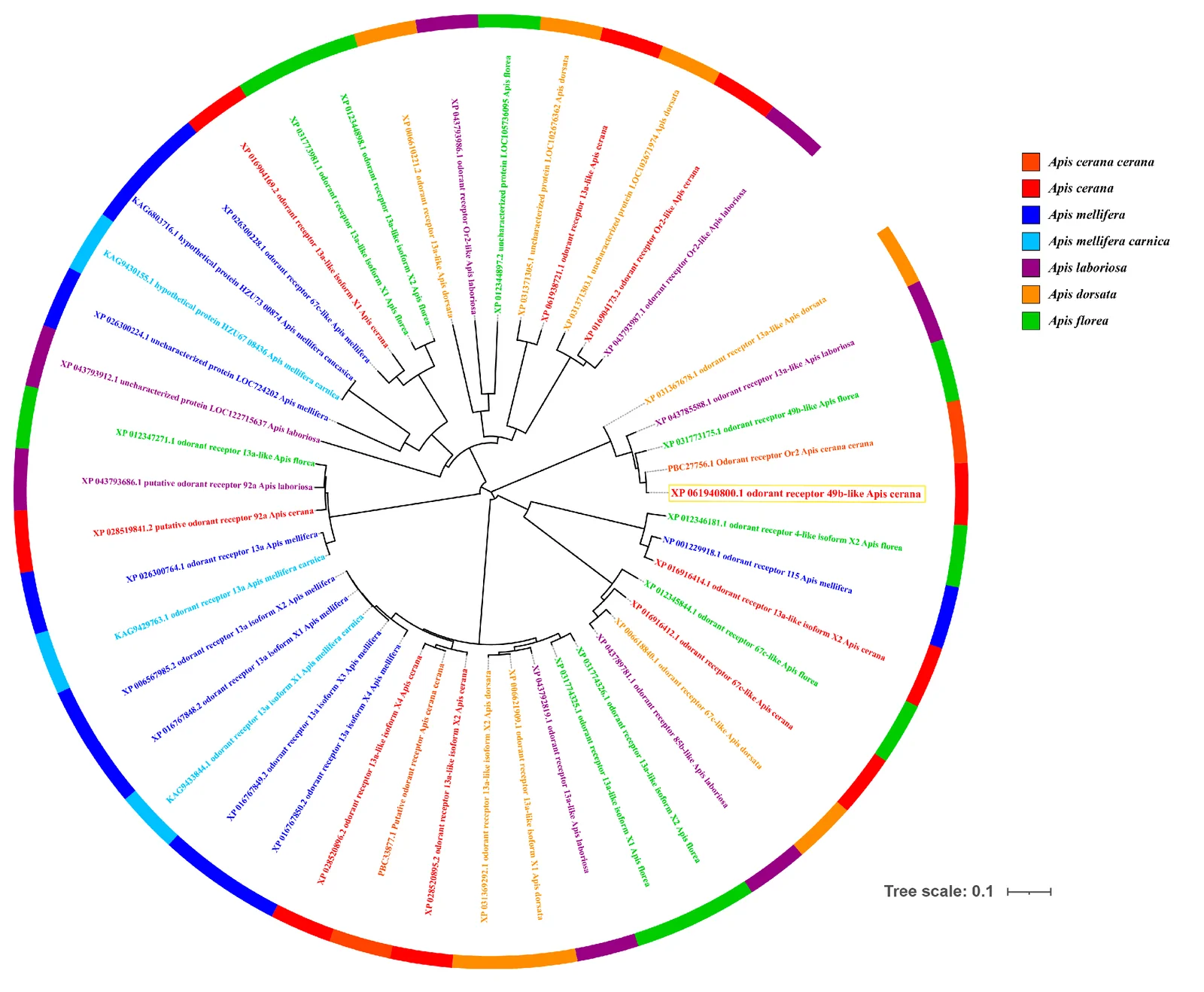
Phylogenetic tree of AcerOR120 and its orthologs in the genus Apis. This tree, constructed based on amino acid sequences of the top 50 BLAST hits homologous to OR120, illustrates the clustering patterns of odorant receptor genes across seven Apis species and their subspecies (*A. cerana*, *A. cerana cerana*, *A. mellifera, A. florea*, *A. dorsata*, *A. laboriosa*, *A. mellifera carnica*, and *A. mellifera caucasica*). Colored fan-shaped regions indicate species-specific clades, and bootstrap values (shown at nodes) represent the statistical support for each branch. The yellow box highlights the odorant receptor investigated in this study.

BLASTp searches retrieved the top 50 amino acid sequences homologous to *AcerOR120* from the genus Apis. Among them, AcerOR120 showed the highest identity of 100% with *Apis cerana* OR49b-like (XP_061940800.1) and *A. c. cerana* OR2 (PBC27756.1); with *Apis florea AfloOR49b-like* (XP_031773175.1) and *Apis laboriosa* OR13a-like (XP_043785588.1), the identities were 94.74% and 94.26%, respectively, indicating that OR120 homologous proteins are highly conserved in the genus Apis. The phylogenetic tree of Apis ORs constructed based on amino acid sequences (Figure 2) showed that all sequences clustered into multiple species-specific branches: *A. cerana* sequences including AcerOR120 and its homologs (PBC33877.1, XP_028520895.2, XP_028520896.2) formed one clade; multiple OR13a homologs from *Apis mellifera* (XP_006567985.2, XP_016767848.2, XP_016767849.2, XP_016767850.2) and its subspecies (*A. mellifera carnica*, *A. mellifera caucasica*) formed another clade; *A. florea* OR49b-like and OR13a-like sequences formed an independent branch; and homologous sequences from *A. laboriosa* and *Apis dorsata* also formed specific branches.

### 3.3. Spatiotemporal Expression Profiles of AcerOR120

**Figure 3:**
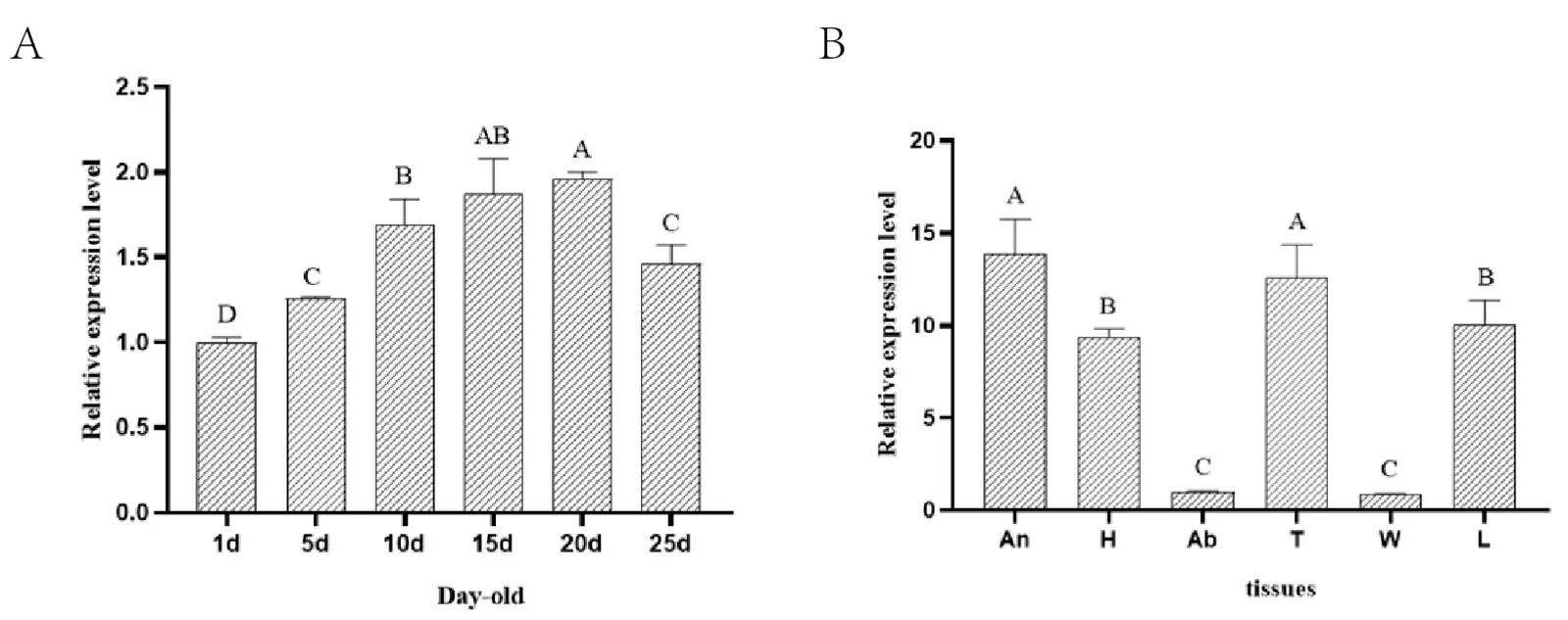
Spatiotemporal expression profiles of *AcerOR120*. (**A**) Relative mRNA expression levels of *AcerOR120* in antennae of worker bees across different developmental stages (1, 5, 10, 15, 20, and 25 days old). (**B**) Relative mRNA expression levels of *AcerOR120* across different tissues of foragers. An: antenna; H: head (without antenna); T: thorax (without wings); Ab: abdomen; L: leg; W: wing. Different capital letters above bars indicate extremely significant differences (*p* < 0.01), while identical letters indicate no significant difference (*p* > 0.05).

The expression of *AcerOR120* in antennae of *A. c. cerana* workers at different developmental stages was analyzed. The results showed that *AcerOR120* was expressed at all ages in worker antennae, and the relative expression levels differed significantly. Overall, expression increased first and then decreased with age: the highest expression was at day 20 and the lowest at day 1. There was no significant difference between day 20 and day 15 (*p* > 0.05), but both were extremely significantly higher than days 1, 5 and 25 (*p* < 0.01). Day 10 was extremely significantly higher than days 5 and 25 (*p* < 0.01). There was no significant difference between day 5 and day 25 (*p* > 0.05), but both were extremely significantly higher than day 1 (*p* < 0.01) (Figure 3A).

Expression in different tissues of foragers was also analyzed. The results showed that *AcerOR120* was expressed in all tested tissues, but the expression abundances differed extremely significantly. The highest levels were found in antennae and thoraces (without wings), with no significant difference between them (*p* > 0.05), and both were extremely significantly higher than other tissues (*p* < 0.01). Heads (without antennae) and legs had intermediate levels, with no significant difference between them (*p* > 0.05). Abdomens and wings had the lowest levels, with no significant difference between them (*p* > 0.05) (Figure 3B).

### 3.4. RNAi Silencing Efficiency

**Figure 4:**
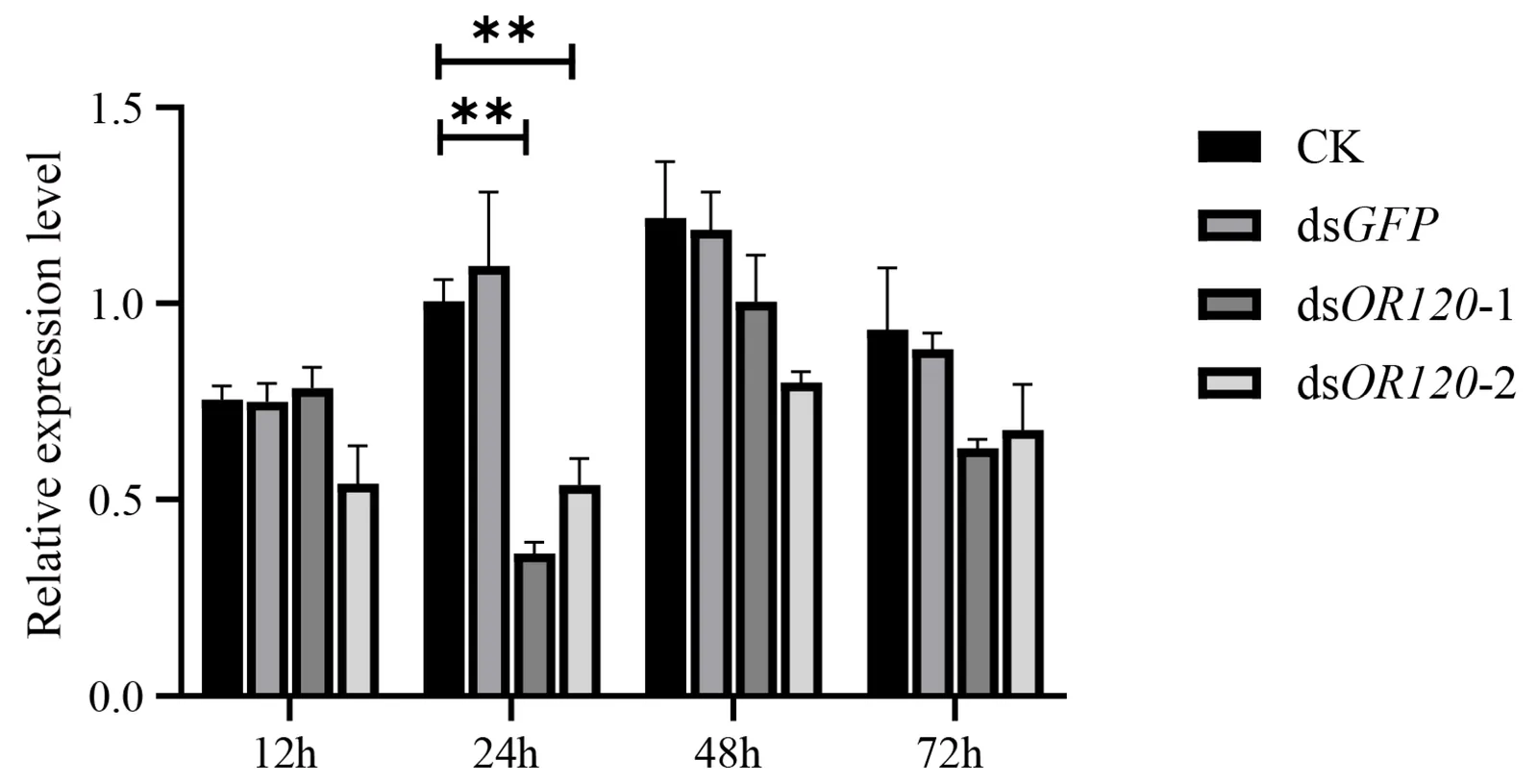
mRNA expression of OR120 at different time points after silencing. ** indicates extremely significant difference between treatments (*p* < 0.01).

Using qPCR, we analyzed the changes in *AcerOR120* expression levels at different time points after feeding different dsRNAs to determine the optimal silencing sequence and time (Figure 4). The results showed that mRNA expression levels in the *dsOR120*-1 and *dsOR120*-2 interference groups at 24 h, 48 h and 72 h were all lower than those in the corresponding CK group and the dsGFP negative control group, and the best interference effect was achieved at 24 h for both. The *dsOR120*-1 sequence had the best silencing efficiency, reaching 63.9%, which was extremely significantly different from the CK group (*p* < 0.01). Therefore, *dsOR120*-1 at 24 h silencing was chosen as the gene silencing treatment for subsequent EAG experiments.

### 3.5. Odor Perception Analysis of AcerOR120

**Figure 5:**
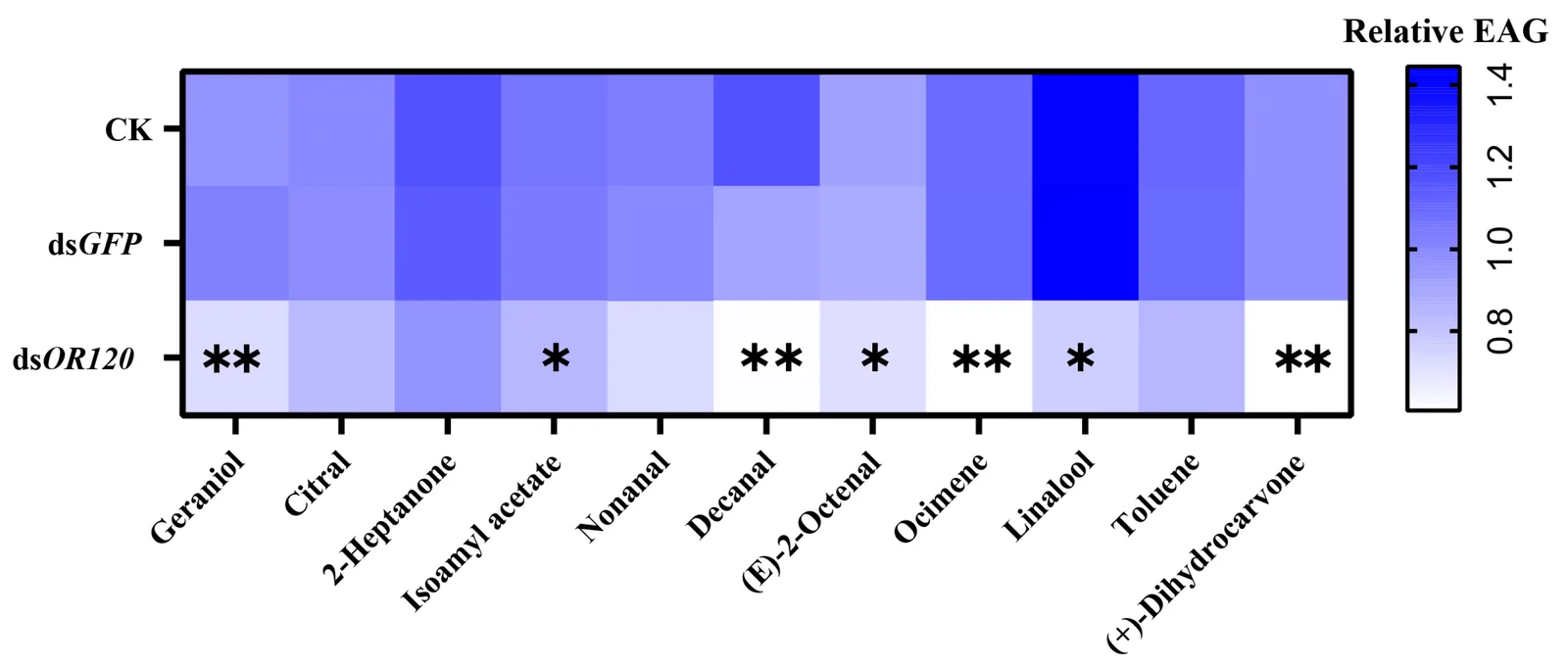
EAG response values of *A. cerana cerana* under OR120 silencing. * indicates extremely significant difference between treatments (*p* < 0.05). ** indicates extremely significant difference between treatments (*p* < 0.01).

EAG values at 300 μg/μL for 11 volatile odorants were measured in CK, *dsGFP* negative control and *dsOR120* interference-treated groups (Figure 5). The results showed that antennae of *A. c. cerana* produced electrical signals in response to all 11 tested volatile compounds under different treatments. Overall, there was no significant difference in EAG values between the CK group and the *dsGFP* group, and the response trends to different odorants were similar, indicating that feeding *dsGFP* had no effect on olfactory sensitivity; thus, the negative control was valid. In contrast, the *dsOR120* interference group showed significantly lower EAG values overall compared to the CK group, with an opposite trend to the control group, indicating that AcerOR120 is related to olfactory sensitivity: as *AcerOR120* expression decreased, the bees’ sensitivity to odors declined.

The EAG responses to different odorants also had their own characteristics. Both CK and dsGFP groups showed relatively high response intensities to 2-heptanone, nonanal and linalool. After *dsOR120* treatment, the most significant decreases in EAG values were observed for geraniol, decanal, ocimene and (+)-dihydrocarvone (*p* < 0.01). Responses to (E)-2-octenal and isoamyl acetate were also reduced to various extents (*p* < 0.05). These results indicate that silencing *AcerOR120* weakens the electrical responses of olfactory neurons in *A. c. cerana* antennae to multiple odor molecules, suggesting that *AcerOR120* plays an important role in the recognition of floral and aliphatic odors in *A. c. cerana*.

## 4. Discussion

The *AcerOR120* ORF is 1203 bp long, encoding 401 amino acids. Combined with protein structure prediction, AcerOR120 possesses 7 transmembrane domains, which matches the typical structural features of insect odorant receptors [17]. Amino acid sequence comparison revealed that AcerOR120 shares high identity with odorant receptors of other Apis insects, especially 94.74% and 94.26% identity with *A. florea* AfloOR49b-like and *A. laboriosa* AlabOR13a-like, respectively, suggesting they may be orthologous genes. It also shows high similarity with ORs from *A. dorsata* and *A. mellifera*. Analysis of the OR gene family closely related to AcerOR120 suggests that despite the sequence conservation of AcerOR120, the limited number of homologous genes available for alignment only allows for the speculation that AcerOR120 may have an early evolutionary origin. Furthermore, this conserved group of ORs likely plays a role in mediating the recognition of basal environmental volatiles and pheromones across bee species [18,19]. Meanwhile, the phylogenetic tree showed that homologous sequences of AcerOR120 formed species-specific branches in multiple bee species, including *A. cerana*, *A. mellifera*, *A. florea* and *A. dorsata*, suggesting that this gene has undergone a long evolutionary history in different bee species, leading to functional diversification and higher value for functional and evolutionary studies [20,21]. Overall, AcerOR120 appears to be an early-originating, highly conserved odorant receptor gene in the genus Apis that generally plays important physiological roles in olfactory recognition of bees.

Gene expression patterns in insects can, to some extent, reflect their biological functions [22]. Generally, sex pheromone receptors are specifically highly expressed in male antennae, while general olfactory receptors have broader expression distributions [23]. In this study, qRT-PCR results showed that AcerOR120 mRNA was most abundant in the antennae of foragers, but also had relatively high expression in heads, thoraces and legs, indicating that AcerOR120 may belong to the general olfactory receptor class. Odorant receptors are expressed in olfactory sensory neurons, and antennae are important olfactory organs of insects, densely covered with olfactory sensilla [3]. Therefore, the high expression of AcerOR120 in antennae is consistent with its predicted olfactory receptor function. In addition, the mouthpart appendages on the insect head also bear numerous sensilla, serving as important sensory and feeding centers [24]. The concurrent high expression across the head, thorax, and legs may enhance the ability of *A. c. cerana* to rapidly respond to complex environments. A similar multi-tissue high expression pattern of ORs was observed in Gryllotalpa orientalis, representing an adaptive evolutionary response to subterranean environments [25]. Meanwhile, the ectopic expression of AcerOR120 suggests potential non-olfactory functions; its abundant expression in the legs implies an involvement in mechanosensory or contact-chemosensory perception, acting similarly to the gustatory receptor for sucrose (AmGr1) to facilitate contact-mediated signaling. It is therefore speculated that AcerOR120 mediates sensory reception across multiple body parts of the honey bee [26,27]. Honey bees are highly social insects, and their biological behaviors are closely related to their developmental stage and environmental changes. Workers have a distinct division of labor according to age. Generally, bees before 18 days old are house bees, mainly responsible for honey ripening, feeding larvae and the queen, cleaning cells, and secreting wax for comb building; after 18 days, they become foragers, mainly responsible for leaving the hive to collect pollen, nectar, water, propolis and guarding the nest [28]. Some studies have shown that in *A. c. cerana* colonies, some workers begin to forage at 12 days old [29]. In this study, we found that AcerOR120 expression in antennae was relatively high at 15–20 days old, suggesting that this gene may be involved in foraging activities. Expression at day 1 was the lowest, possibly because the bees had just emerged and their physiological functions were not fully developed, so olfactory-related gene expression was also low. The decline at day 25 may be because the bees had entered a stable foraging period, and also because high-intensity foraging activities might weaken olfactory perception, with the underlying change being decreased expression of olfactory genes and reduced olfactory sensitivity [30,31].

Through EAG analysis of 11 odorants before and after silencing *AcerOR120*, we found that silencing the gene reduced olfactory sensitivity to most odorants, indicating that *AcerOR120* may be involved in the recognition of multiple odor signals. The recognition of different odors by bees affects their functional performance. Among them, geraniol, decanal and (+)-dihydrocarvone are common floral volatiles [32]; reduced sensitivity to these floral odors after silencing *AcerOR120* may be involved in the peripheral perception of floral fragrance cues. Ocimene is a larval pheromone that influences worker brood care behavior and also inhibits worker ovarian development, maintaining colony stability [33]. The decreased EAG response to ocimene following OR120 silencing implies a potential capability of this receptor to detect colony-internal chemical signals, although its exact physiological role remains to be elucidated. In summary, OR120 is a typical odorant receptor highly expressed during the foraging period and involved in olfactory activities of *A. c. cerana*. However, the current results on *AcerOR120* are still insufficient to fully explain its specific functions, and the above inferences await further verification. Constrained by current experimental approaches, these findings offer only preliminary insights into the in vivo functions of *AcerOR120*. Future studies employing heterologous expression systems—such as Xenopus oocytes or HEK293 cells—are needed to provide direct functional validation and characterize its ligand specificity, thereby establishing a stronger theoretical and empirical basis for the practical applications of *AcerOR120*.

## Figures and Tables

**Table 1:** Primers for qRT-PCR.

Gene Name	Primer Sequences (5′-3′)	Gene Name	Primer Sequences (5′-3′)
AcerOR120 -F	TTCCATTTGCTGCATCATATCC	AcerOR120-R	TTGCAAGCATCATCAGACAAC
Arp1-F	ACTACGGCCGAACGTGAAAT	Arp1-R	GGAAAAGAGCCTCGGGACAA
AcerOR120-T1-dsF	taatacgactcactatagggTTGTTGTCTGATGATGCTTGC	AcerOR120-ds2-R	AGAAATCTGAAATCCGACGAAAC
AcerOR120-ds2-F	TGTTATGGAAACGGGCTCAAC	AcerOR120-T2-dsR	taatacgactcactatagggAGAAATCTGAAATCCGACGAAAC
AcerOR120-T2-dsF	taatacgactcactatagggTGTTATGGAAACGGGCTCAAC	AcerOR120-ds1-R	TGCAGTACTAAGTATCGCGG
GFP-ds2-F	CACAAGTTCAGCGTGTCC	GFP-T2-dsR	taatacgactcactatagggGGGCACCGCAATAGGAGC
GFP-T1-dsF	taatacgactcactatagggCACAAGTTCAGCGTGTCC	GFP-ds1F-R	CTGGGTGCTCAGGTAGTG

**Table 2:** Odorant material used in this study.

Odorant Compounds	CAS No.	SG	Purity	Manufacturer
Geraniol	106-24-1	0.88	96%	Aladdin (Shanghai, China)
Citral	5392-40-5	0.82	94%	Aladdin (Shanghai, China)
2-Heptanone	110-43-0	0.82	98%	Aladdin (Shanghai, China)
Isoamyl acetate	123-92-2	0.87	>98%	Aladdin (Shanghai, China)
Nonanal	124-19-6	0.83	>95%	Aladdin (Shanghai, China)
Decanal	112-31-2	0.83	>97%	Aladdin (Shanghai, China)
Ocimene	13877-91-3	0.8	>90%	Aladdin (Shanghai, China)
(E)-2-Octenal	2548-87-0	0.846	95%	Aladdin (Shanghai, China)
(+)-Dihydrocarvone	7764-50-3	0.929	98%	Aladdin (Shanghai, China)
Toluene	108-88-3	0.872	99.80%	Aladdin (Shanghai, China)
Linalool	78-70-6	0.86	98.50%	Aladdin (Shanghai, China)

Note: SG stands for Specific Gravity (relative density), which represents the ratio of the density of the odorant to the density of water. The SG values were provided by the manufacturer based on standard technical specifications (typically measured at 20 °C or 25 °C).

## Data Availability

The data presented in this study are available from the corresponding author upon reasonable request.
